# Supplementary material for: Hair Thickness Growth Effect of Adenosine Complex in Male-/Female-Patterned Hair Loss via Inhibition of Androgen Receptor Signaling
Source: Int J Mol Sci. 2024 Jun 13;25(12):6534. doi: 10.3390/ijms25126534 (PMC11204140; doi:10.3390/ijms25126534)
Supplement: Supplementary file 1 [file ijms-25-06534-s001.zip › ijms-3001581-supplementary.pdf]

Supplemental Information

Supplementary Figure S1. mRNA expression profile by minoxidil and adenosine in dermal papilla cell

mRNA analysis of human dermal papilla cell with minoxidil and adenosine treatment. Relative expression with non-treated control as 100%. We examined 78 genes with 6 categories; Cell differentiation, Cell junction, and Cellular survival, Cytoskeleton, Growth factor signaling, and Tissue development. The expression level of 41 genes were significantly changed by adenosine or minoxidil.

	MNX	Adenosine	
	100nM	1.5mM	3mM
IGF1	142%	346%	487%
KDR	65%	159%	246%
FZD4	184%	195%	194%
EGF	103%	166%	178%
FGF2	147%	127%	168%
FZD5	123%	128%	159%
FGF7	143%	153%	156%
DCN	127%	154%	149%
FZD3	81%	124%	149%
KI67	140%	143%	148%
FGFR1	148%	142%	138%
ITGB1	136%	141%	135%
VEGFA	167%	135%	133%
BCL2	129%	117%	130%
LEF1	108%	128%	129%
CTNNB1	115%	134%	128%
PDGFRA	90%	133%	122%
WNT5A	138%	140%	118%
FZD7	79%	97%	104%
CASP9	80%	97%	96%
CASP3	83%	96%	84%
GSK3B	103%	84%	82%
BMP2	85%	74%	79%
BAD	58%	84%	75%
ROR2	90%	71%	70%
DKK1	66%	73%	63%
PDGFRB	68%	68%	59%
TGFB1	56%	48%	57%
CCND1	57%	51%	55%
VCAN	65%	65%	54%
LRP5	71%	65%	54%
NCAM1	81%	51%	54%
CDKN1A	70%	81%	52%
AXIN2	99%	82%	50%
BMP4	84%	66%	50%
ROR1	49%	53%	47%
IGFBP2	71%	67%	47%
FZD7	85%	67%	44%
FLT1	99%	66%	42%
CDKN2A	77%	53%	39%
TGFB2	56%	47%	39%

A) Cell Differentiation [ALPL (Alkaline Phosphatase), CREB1 (CREB), Myc (c-Myc), PTEN (PTEN), SOX13 (Sox13), SOX2(Sox2), SOX9 (Sox9), TEAD1 (TEF1)]

B) Cell Junction [DCN (Decorin), CDH1 (E-cadherin), CDH2(N-cadherin), CDH3 (P-cadherin), ITGA5 (Integrin a5), ITGB1 (Integrin b1), NCAM1 (NCAM), VCAN (Versican), VIM (Vimentin)]

C) Cell Survival [BAD (Bad), BAX (Bax), BCL2 (Bcl2), CASP3 (Caspase-3), CASP9 (Caspase-9), CDK1(Cyclin-dependent kinase 1), CDK2 (Cyclin-dependent kinase 2), CDK4 (Cyclin-dependent kinase 4), CCNA2 (Cyclin A2), CCNB1 (Cyclin B1), CCND1 (Cyclin D), MKI67 (Ki67), MDM2 (MDM2), CDKN1A (p21), CDKN2A (p16), TP53 (p53)

D) Cytoskeleton [CD200 (CD200), Col1A1 (Collagen type 1 alpha1), ENG (Endoglin(CD105)), FN1(Fibronectin), NES (Nestin), KRT14 (Keratin 14)]

E) Growth Factor Signaling [FGF2 (bFGF), EGF (EGF), EGFR (EGF Receptor), FGF10 (FGF10), FGFR1(FGF receptor 1), FGFR2 (FGF Recetpor 2), IGF-1(IGF-1), IGFBP2 (IGF Binding Protein 2), FGF7 (KGF), PDFGA (PDGF a subunit), PDGFB (PDGF b subunit), PDGFRA (PDGF Receptor A), PDGFRB (PDGF Receptor B), TGFb1 (TGF beta 1), TGFb2 (TGF beta 2), VEGFA (VEGF-A), KDR (VEGF Receptor 2)

F) Tissue Development [AXIN2 (Axin), CTNNB1 (beta-catenin), BMP2 (BMP2), BMP4 (BMP4), BMPR1A (BMP Receptor 1), DKK1 (DKK-1), EDAR (EDAR), FOXN1 (Foxn1), FZD3 (Frizzled 3), FZD4 (Frizzled 4), FZD5 (Frizzled 5), FZD7 (Frizzled 7), GSK3B (GSK3 beta), LEF1 (Lef1), LRP5 (Lrp 5), LRP6 (Lrp6), NOG (Noggin), NOTCH1 (Notch1), ROR1 (Ror1), ROR2 (Ror2), WNT5A (Wnt-5a), WNT10B (Wnt-10b)

Supplementary Table S1: GO analysis: Activated pathways by both of MNX and Adenosine

#pathway ID	pathway description	observed gene count	p-value
GO.0042325	regulation of phosphorylation	24	3.19×10^{-18}
GO.0008284	positive regulation of cell proliferation	21	4.24×10^{-18}
GO.0032270	positive regulation of cellular protein metabolic process	23	4.32×10^{-17}
GO.0001932	regulation of protein phosphorylation	22	1.08×10^{-16}

GO.0050678	regulation of epithelial cell proliferation	15	1.08×10^{-16}

Supplementary Table S2: GO analysis: Activated pathways by Minoxidil

#pathway ID	pathway description	observed gene count	p-value
GO.0007166	cell surface receptor signaling pathway	13	1.82×10^{-8}
GO.0048646	anatomical structure formation involved in morphogenesis	11	1.82×10^{-8}
GO.0048010	vascular endothelial growth factor receptor signaling pathway	6	2.20×10^{-7}
GO.0072358	cardiovascular system development	9	5.54×10^{-7}
GO.0001568	blood vessel development	7	4.94×10^{-6}

Supplementary Table S3: GO analysis: Activated pathways by Adenosine

#pathway ID	pathway description	observed gene count	p-value
GO.0048568	embryonic organ development	13	5.06×10^{-13}
GO.0022603	regulation of anatomical structure morphogenesis	15	5.91×10^{-13}
GO.0048732	gland development	12	2.09×10^{-12}
GO.0072358	cardiovascular system development	14	2.32×10^{-12}
GO.0051094	positive regulation of developmental process	15	3.63×10^{-12}

Supplementary Table S4: Participant Statistics

For evaluation of hair thickness, Korean alopecia cohort (n=156) were investigated.

	Hamilton Nordwood Scale				
	I	II	III	IV	V - VI
Number of Volunteers	14	27	45	42	28
Average Ages	33.57	35.30	42.22	47.36	46.68
Hair Density (number/cm2)	189.05	169.07	173.74	164.25	177.01
Hair Shaft Thickness (μm)	71.3	59.7	57.7	53.4	47.8
Average hair thickness counting number for person (number/person)	81.14	76.78	86.49	84.69	92.46

Supplementary Table S5: Hair thickness evaluation following by sampling number

Measuring accuracy of hair shaft thickness following by sampling number of shafts. Statistically significant with each measured value * $p < 0.05$

		Hair Shaft Thickness (μm)								
		Measured Value	Random sampled Evaluation							
			5 Samples	10 Samples	15 Samples	20 Samples	30 Samples	40 Samples	50 Samples	60 Samples
Hamilton -Norwood Scale	I	71.3	61.3*	69.1	69.7	72.2	71.2	71.2	71.9	71.8
	II	59.7	54.0*	57.3*	57.8*	60.2	59.6	60.4*	60.0	60.3
	III	57.7	51.5*	55.2*	57.4	58.4	56.8*	58.0	57.5	57.9
	IV	53.4	48.1*	51.8*	52.3*	52.2*	53.2	53.8	53.8	53.7
	V - VI	47.8	47.1	46.3*	46.8	47.3	47.7	48.0	47.8	48.0

Supplementary Table S6: Participant Statistics for administration of minoxidil and adenosine complex

The hair thickness enhancement by minoxidil and adenosine complex treatment. For evaluation of effect MNX and APN on hair thickness, topical administration to Korean alopecia cohort (n=46) were investigated.

		Group 1 (MNX)		Group 2 (APN)	
Total Number of Volunteers		26		20	
Min. age	[years]	30		25	
Max. age	[years]	61		53	
Mean age	[years]	46.5	± 9.1	47.5	± 8.6
Average of Hair Density (± STD)	[number/cm^2]	168.1	± 21.8	161.3	± 22.7
Hamilton-Norwood Scale [Number]	II	5		3	
	III	6		8	
	IV	8		6	
	V	6		3	
